# Books and Pamphlets Received

**Published:** 1888-06

**Authors:** 


### BOOKS AND PAMPHLETS RECEIVED.

*A Year's Work in Abdominal Surgery*, with a Report of Eighty Laparotomies done in 1887. By W. Gill Wylie, M D., New York.

*One Hundred and Ten Laparotomies* for the Removal of the Uterine Appendages. By Professor W. Gill Wylie, M. D., New York.

*Clinical Notes on Pruritus.* By Duncan Bulkley, A. M., M. D., attending Physician to the New York Skin and Cancer Hospital, etc.

*Papillomatous Cystic Tumor of Ovary*, with a Hernial Pouch Developed in the Cicatrix of the Abdominal Wound from a former Ovariotomy. By L. H. Laidley, M. D., Professor of Gynecology, Beaumont Medical College; Surgeon to Protestant Hospital; Consultant to St. Louis Female Hospital. Read before the St. Louis Medical Society, March 10, 1888.

*Infantile Feeding;* especially with reference to subjects with infantile eczema. By L. Duncan Bulkley, A. M., M. D.; Physician to New York Skin and Cancer Hospital, etc., read in the Section on Diseases of Children, at the Thirty-eighth Annual Meeting of the American Medical Association, June, 1887.

*Chemical Analyses of Healthy and Diseased Urine, Qualitative and Quantatative.* By T. C. Van Nuys, Professor of Chemistry, Indiana University; with thirty-nine wood engravings. Philadelphia: P. Blakeston, Son & Co., 1012 Walnut st. 1888.

The above is a plain, practical, illustrated book, well written and comprehensive, adapted to both students and practitioners. It contains 187 large octavo pages neatly bound in cloth, in large excellent type.

*Earth as a Topical Application in Surgery;* being a fair exposition of its use in all the cases requiring topical applications admitted in the Men's and Women's Surgical Wards of the Pennsylvania Hospital during a period of six months. By Addenell Hewson, M.D., second edition, with four photo relief illustrations. Philadelphia: The Medical Register Co. 1887.

This work contains many cases illustrative of the beneficial effects of dry earth as a topical application to wounds, burns, ulcerated surfaces of various kinds. The book contains 300 pages, and is worthy of perusal.

*The Surgical Diseases of the Genito-Urinary Organs including Syphilis.* By. E. L. Keyes, A.M., M.D., Professor of Genito-Urinary Surgery, Syphilology and Dermatology in Bellevue Hospital Medical College; Consulting Surgeon to the Charity, the Bellevue and the Skin and Cancer Hospital; Consulting Surgeon to the Bureau of Out-Door Relief Bellevue Hospital; A Revision of Van Buren and King's Text-Book upon the same subjects. New York: D. Appleton & Co. 1888. 705 large octavo pages. Price $5.00.

We have here a practical work, the material of which is drawn largely from cases occuring in private and hospital practice, by those who have made special study and observation in urinary diseases. The author has also made careful investigation into the literature of the subject, and has reproduced the more practical facts of the same in such concise manner that the reader may readily avail himself of them. The consideration of syphilis, in connection with urinary troubles, is a good feature in the present work as success in the treatment of this class of affections largely depends on a knowledge of syphilitic complications.

*The Rectum and Anus; their Diseases and Treatment.* By Charles B. Ball, M. Ch., University Dublin, F. R. C. S. I.; Surgeon to Sir Patricks Dun's Hospital; University Examiner in Surgery, and Member of Surgical Court of Examiners, Royal College of Surgeons, Ireland. With fifty-four illustrations and four colored plates. Philadelphia: Lea Brothers & Co , Publishers. Price, $2.25.

The author excuses himself for adding this book to the voluminnious literature now existing on rectal diseases, on the ground that recent improvements have produced new and important changes in this as in all departments of surgery. These improvements have modified our views of treatment in many essential particulars. The present work aims to present these improvements, thus bringing the subject well up to the highest advances made. The book is well written, and contains a number of beautiful colored illustrations. We are favorably impressed with the work as calculated to add new and advanced suggestions in the nature and treatment of hemorrhoidal affections.

*Rules of Aseptic and Antiseptic Surgery;* a Practical Treatise for the use of Students and the General Practitioner. By Arpard G. Gerster, M. D., Professor of Surgery, at the New York Polyclinic; Visiting Surgeon to Mt. Sinai Hospital, and the German Hospital, New York. Illustrated with 248 engravings, and three cromo-lithographic plates. New York: D. Appleton & Co. 1888.

A work containing 332 large octavo pages, neatly bound in cloth and beautiful in typographical execution. It gives a full systematic and practical presentation of the antiseptic treatment in surgery in accordance with the principal introduced by Lester. Whatever difference of opinion may exist as to the correctness of the theory of Lester, the fact is patent that a great improvement has occurred in the results of surgical practice under the antiseptic methods explained and illustrated in this work. The author draws largely from his own personal experience in the facts presented. The illustrations are excellent and from life, by photographic process, and the entire work is one of marked ability. It should be in the library of every surgeon.

*A Practical Treatise on Diseases of the Skin.* By John V. Shoemaker, A. M., M. D., Professor of Skin Diseases in the Medico Chirurgical College and Hospital, of Philadelphia; Physician to the Philadelphia Hospital for Diseases of the Skin ; Member of the American Medical Association, etc., etc., with colored plates and other illustrations. New York: D. Appleton & Co. 1888.

This is a large, able and comprehensive work containing 633 large octavo pages, neatly bound in cloth. Price, $5.00.

The work is written from the stand point of an active practitioner, and is eminently practical and suggestive. The illustrations are beautiful.

The subjects are considered under the following heads and captions: General Considerations; Disorders of Secretions; Hyperæmeas, Hemorrhages; Exudations; Hypertrophies, Atrophies; Tumors; Neurosis; Parasites; Formulary.

The book is well suited to bo h student and practitioner. It is neatly bound on c'oth. Price, $5.00.

*Lindsay & Blakiston Visiting List for* 1888. This is excellent—among the very best. Prices vary from $1 to $3, according to the size and number of patients. Adapted to twenty-five to one hundred patients. This List is well known to the profession, having now reached its 37th edition. Address P. Blakiston, Son & Co., 1012 Walnut street.

*Evacuant Medication—Cathartics and Emetics.* By Henry M. Field, M.D. Professor of Therapeutics Dartmouth Medical College; Corporate Member of New York Academy of Medicine. Philadelphia: P. Blakiston, Son & Co, 1012 Walnut street. 1887. 288 pp. Price. $1.75.

We find this a valuable work and recommend it to the profession as containing many instructive and practical suggestions from an able and experienced teacher in therapeutics.

*Operative Surgery on the Cadav r.* By Jasper Jewett Garmony, Attending Surgeon to Out-door Poor Dispensary of Bellevue Hospital; Visiting Surgeon to Ninety-ninth Street Reception Hospital, branch of Bellevue Hospital; Member of the British Medical Association, etc. New York: D. Appleton & Co. 1887.

The above is a very useful and practical work of 150 octavo pages. In which are ably treated "The Use of Specula, Catheters, Sounds, Bougies," etc. "Paracentesis, Hypodermic Needle," etc.; "Manipulation of the Scalpel," etc.; "Operations on the Trunk," etc.; "Genito Vesical Operations;" Operations on Muscles," "Operations on Nerves," "Operations on Circulatory System," "Operations on Osseous System," "Amputations and Disarticulations," etc.

*Practical Lessons in Nursing—Fever Nursing.* Designed for the use of professional and other nurses and especially as a text book for nurses in training. By J C. Wilson, A.M., M.D, author of a treatise on "Continued Fevers;" Visiting Physician to the Philadelphia Hospital and to the Hospital of the Jefferson College; Fellow of the College of Physicians, Philadelphia, etc. Philadelphia: J. B. Lippincott Company. 1888. 12 mo. 200 pages, extra cloth. Price, $1.00.

We regard this little work as very practical and useful, not only to the nurse, but to the practicing physician.

*Doctor and Patient.* By S. Weir Mitchell, M.D., L.L.D., Member of the United States National Academy of Sciences; President of the College of Physicians of Philadelphia; Physician to the Orthopædic Hospital and Infirmary for Nervous Diseases. Philadelphia: J. B. Lippincott Company. 1888. p. 177; Price, $1.50

Embraces the following chapters: 1. Introductory. 2. The Physician. 3. Convalescence. 4. Pain and its consequences. 5. The Moral Management of Sick and Invalid Children. 6. Nervousness and its Influence on Character; 7. Out-door and Camp Life for Women.

The work is well and ably written, and contains much that is interesting, useful and practical.

